# Mit Strom gegen den Kopfschmerz: Die Elektro- und tDCS-Therapie der 1870er-/1880er-Jahre und von heute

**DOI:** 10.1007/s00482-023-00746-1

**Published:** 2023-08-24

**Authors:** Zhenya Wagner, Holger Steinberg

**Affiliations:** Forschungsstelle für die Geschichte der Psychiatrie, Klinik und Poliklinik für Psychiatrie und Psychotherapie, Medizinische Fakultät, Universität Leipzig, Semmelweisstr. 10, 04103 Leipzig, Deutschland

**Keywords:** Analgesie, Migräne, tDCS, Elektrotherapie, Geschichte der Schmerztherapie, Analgesia, Migraine, tDCS, Electric stimulation therapy, History of pain therapy

## Abstract

Kopfschmerzen sind sowohl ein verbreitetes Symptom als auch oft genug in sich ein Erkrankungsbild, das zu Leidensdruck, Behinderung und gesamtökonomisch hohen Kosten führt. Die medikamentöse Therapie wirkt oft nur unzureichend oder bringt andere Limitationen mit sich. Die Anwendung von Strom erschien bereits im 19. Jahrhundert eine vielversprechende Behandlungsmethode zu sein und auch aktuell wird zur Anwendung der tDCS bei dieser Indikation geforscht. Diese Arbeit gibt eine Übersicht sowohl über die während der ersten Blütezeit der Elektrotherapie Ende des 19. Jahrhunderts als auch über die in der kontemporären Forschung erschienenen Studien, die sich mit der Anwendung schwacher elektrischer Ströme zur Therapie oder Prophylaxe von Kopfschmerzen auseinandersetzen. Es zeigt sich, dass vorrangig vielversprechende Behandlungserfolge präsentiert werden, wobei die Fallzahlen oft gering und die eingesetzten Stimulationsmethoden sehr heterogen sind. In Summe scheint die elektrotherapeutische Anwendung zur Therapie von Kopfschmerzsyndromen ein auf eine lange Tradition zurückblickender, interessanter Forschungszweig und Therapieansatz zu sein, wobei noch weitere Forschung notwendig ist, sowohl bezüglich der technischen und klinischen Details der Durchführung der Stimulation als auch hinsichtlich der verschiedenen Indikationen.

## Abstract (en)

Headache can be a widespread symptom as well as a disorder in itself. Headache syndromes such as migraine cause a lot of distress, disability and overall socioeconomic costs. Pharmacological treatments are often limited in their efficacy as well as due to side effects. The therapeutic application of electricity for this medical indication was a relevant field of research in the 19th century and—in the form of transcranial direct current stimulation (tDCS)—is still widely studied today. This paper provides an overview of publications from the late 19th century (as the era of discovery and success of electrotherapy) as well as contemporary studies investigating the usage of weak currents for the treatment or prophylaxis of headache. Our results show a large number of highly favorable reports of treatment successes. However, the number of cases analysed is often rather small and the forms of electric stimulation applied were often highly heterogeneous. In summary, electric stimulation appears to be a promising field of research and a possible therapeutic agent for the treatment of headaches; however, further research is necessary, especially into the details of the stimulation techniques applied and the various indications in which it may be of use.

Trotz ihrer Verbreitung sind Kopfschmerzen immer noch ein Phänomen, dessen Therapie sich oft schwierig gestaltet. Dabei blickt die Anwendung schwacher Ströme als Therapeutikum auf eine lange, wenn auch unterbrochene Forschungstradition zurück und scheint ein erforschens- und beobachtenswerter Ansatz zu sein. Für eine abschließende solide wissenschaftliche Aussage über den unzweifelhaften Erfolg liegen aber nach wie vor zu wenige korrelative Studien und damit verlässliche Daten vor. In dieser Arbeit werden wissenschaftliche Publikationen zur Elektrotherapie aus der 2. Hälfte des 19. Jahrhunderts und zeitgenössische Studien zur Anwendung von transkranieller Gleichstromstimulation (tDCS) bei Kopfschmerzen identifiziert, gegenübergestellt und diskutiert.

## Hintergrund

„Der Schmerz ist das wichtigste aller Krankheitssymptome, er treibt den Kranken zum Arzt und dieser soll vor allem Schmerzen vertreiben oder lindern“, schrieb der Nervenarzt Paul Julius Möbius 1880 [27, S. 501], und diese Beobachtung bleibt bis heute gültig. Kopfschmerzen sind ein häufiges Symptom und oft auch bereits in sich ein Erkrankungsbild. Mit einer 1‑Jahres-Inzidenz von 46 % und einer Lebenszeitprävalenz von 64 % [24] gehören Kopfschmerzen zu den verbreitetsten gesundheitlichen Beschwerden. Kopfschmerzsyndrome stellen eine relevante Ursache für Behinderung dar [46] und verursachen zwar nicht vorrangig durch ihre Behandlung, aber insbesondere durch Arbeitsausfälle und geringere Produktivität erhebliche Kosten [20]. Zudem sind sie häufig mit Angst- und depressiven Symptomen assoziiert [19] und korrelieren oft mit Arbeitslosigkeit oder geringem Einkommen [8]. Die pharmakotherapeutische Behandlung mit akut wirksamen Medikamenten (z. B. nichtsteroidale Antirheumatika (NSAR) wie Ibuprofen, Acetylsalicylsäure und Naproxen oder Triptane) ist oft durch unerwünschte Arzneimittelwirkungen bei längerer Einnahme sowie die Entwicklung eines medikamentös induzierten Kopfschmerzes bei häufigem Gebrauch [49] limitiert. Zudem ist beispielsweise bei Migräne nur bei etwa der Hälfte der Patienten die akute analgetische Wirkung zufriedenstellend [7]. Die medikamentöse Prophylaxe von Kopfschmerzepisoden ist oft ebenfalls mit erheblichen unerwünschten Arzneimittelwirkungen [35] und einer geringen Adhärenz von Patientenseite [6] verbunden. Verhaltenstherapeutische Ansätze in der Behandlung von Kopfschmerzsyndromen sind oft auch durch mangelnde Patientenadhärenz und geringe Bereitschaft zur Inanspruchnahme in ihrer Wirksamkeit begrenzt [39].

Vor diesem Hintergrund ist es wenig überraschend, dass nichtmedikamentöse somatische Therapieverfahren bei Kopfschmerzsyndromen sich eines großen Forschungsinteresses erfreuen. Ein Verfahren, das in den letzten 20 Jahren vielfach untersucht wurde, ist die tDCS. Sie ist in ihrer Anwendung kostengünstig, komplikationsarm und stellt einen Hoffnungsträger in der Behandlung verschiedenster neuropsychiatrischer Erkrankungen dar. In den kontemporären Veröffentlichungen bleibt jedoch meist unerwähnt, dass dieses Verfahren auf eine über 140-jährige medizinwissenschaftliche Tradition zurückblickt [44]. Bereits in den 1870er-/1880-Jahren erlebte unter der Bezeichnung „Elektrotherapie“ die transkutane Anwendung von schwachen elektrischen Strömen einen regelrechten Boom in der Humanmedizin, vorrangig bereits damals bei neurologischen und psychiatrischen Erkrankungsbildern, jedoch auch bei Stoffwechselstörungen (Diabetes, Gicht, Morbus Basedow), gynäkologischen Erkrankungsbildern und sogar Tumoren.

Aufgrund der oft von den heutigen Bezeichnungen abweichenden historischen Terminologie und damals weitestgehend fehlenden Standardisierung ist es schwierig, eine genaue Darstellung der Ende des 19. Jahrhunderts verwendeten Technologien zur Durchführung der Elektrotherapie zu geben. Grundlegend fanden drei Arten der Anwendung von elektrischem Strom im Rahmen der Elektrotherapie der zweiten Hälfte des 19. Jahrhunderts Verwendung: Galvanisation, Franklinisation und Faradisation. Hierbei war in Summe die Galvanisation bei neurologischen und psychiatrischen Indikationen das am weitesten verbreitete Verfahren [45].

Im Rahmen der Galvanisation wurde ein schwacher, aber hochgespannter Gleichstrom angewendet. Der Strom wurde hierbei mithilfe von galvanischen Elementen – in der Regel Zink-Kohlenstoff-Zellen, manchmal auch Zink-Kupfer-Zellen – erzeugt und über zwei Elektroden, die in der Regel aus einem Zink- und einem Kupferblech bestanden und über einen übersponnenen Kupferdraht miteinander verbunden waren, in den menschlichen Körper geleitet. Bei der Franklinisation erfolgte die Behandlung mittels statischer Elektrizität, die mithilfe des Phänomens der Influenz (oder auch elektrostatischen Induktion) durch eine sogenannte Influenzmaschine generiert wurde. Die Faradisation dagegen setzte keinen konstanten, sondern einen unterbrochenen, rasch pulsierenden Gleichstrom ein, der im physikalischen Sinne einem unsymmetrischen Wechselstrom entspricht. Die Erzeugung des Stroms erfolgte durch elektrische Induktion, sodass auch die hierbei Verwendung findende Technologie die Bezeichnung Induktionsapparat erhielt [45].

Ziel dieser Arbeit ist es, eine Übersicht über die historischen wie auch kontemporären Studien, die sich mit der Anwendung schwacher elektrischer Ströme zur Therapie und/oder Prophylaxe von Kopfschmerzen befassen, zu geben sowie Parallelen und Unterschiede hinsichtlich ihrer Indikationen, Stimulationsmethodiken und Behandlungserfolge herauszuarbeiten. Hierbei handelt es sich nicht um eine systematische, methodisch strikt umgesetzte Metaanalyse, vielmehr ist der Anspruch dieses Artikels, auf die historischen Kontinuitäten, aber auch Entwicklungen in der Anwendung elektrotherapeutischer Verfahren in der Behandlung von Kopfschmerzen aufmerksam zu machen.

## Studiendesign und Untersuchungsmethoden

Im Rahmen dieser Arbeit wurden wissenschaftliche Kasuistiken und Studien zur Anwendung von schwachen elektrischen Strömen bei Kopfschmerzen, die während der ersten Hochphase der Elektrotherapie in der 2. Hälfte des 19. Jahrhunderts erschienen, identifiziert und hinsichtlich der spezifischen Aspekte der Indikation, der angewendeten Behandlung und des Behandlungserfolgs ausgewertet. Zudem wurden kontemporäre wissenschaftliche Publikationen über die Anwendung der tDCS bei Kopfschmerzen recherchiert und ebenfalls im Hinblick auf die Stimulationsformen und das Behandlungsoutcome analysiert.

Ausgangspunkt für die Literaturrecherche zur historischen Elektrotherapie waren die Bände der Rezensionszeitschrift „Schmidts Jahrbücher der in- und ausländischen gesammten Medicin“ der Jahre 1882 bis 1901. Hierin erschien alle paar Jahre die Sammelbesprechung „Ueber neuere elektrotherapeutische Arbeiten“, in der alle dem Rezensenten in den vorherigen Jahren bekannt gewordenen (dadurch – wie z. B. 1882 – zum Teil auch einige Jahre in ihrer Erstveröffentlichung zurückliegenden) Publikationen zum Thema aufgelistet und zum Teil auch kurz inhaltlich dargestellt und beurteilt wurden. In den Jahren 1882, 1884, 1887, 1888 und 1891 zeichnete der bekannte Leipziger Nervenarzt und Wissenschaftspublizist Paul Julius Möbius für diese Sammelbesprechungen verantwortlich. Im Jahre 1897 besprach der Uchtspringer Anstaltsdirektor Konrad Alt und 1901 der niedergelassene Leipziger Nervenarzt Franz Winterscheid die betreffende Literatur. Alle in diesen Jahren aufgelisteten Kasuistiken oder Studien, die sich mit der elektrotherapeutischen Behandlung von Kopfschmerzen – sei es als alleinstehendes Krankheitsbild oder als prominentes Symptom im Rahmen einer weiteren Erkrankung – befassten, wurden im Original in Gänze inhaltlich erfasst, analysiert und einander in wichtigen Eckpunkten gegenübergestellt (Tab. 1).

**Tab. 1:** Übersicht über die in der 2. Hälfte des 19. Jahrhunderts erschienenen Publikationen zur Elektrotherapie bei Kopfschmerz

(Erst‑)Autor	Jahr	Probandenzahl	Indikation	Art der Stimulation	Dauer der Stimulation	Frequenz/Häufigkeit der Stimulation	Kontrollgruppe/Verblindung	Erfolg	Zitat
Katyschew	1878	Keine Angabe	Congestiver Kopfschmerz	Faradisation des Plexus caroticus	Keine Angabe	Keine Angabe	Keine	Rasche und lang anhaltende Effekte	[18]
Calonius	1879	4 (2 w)	Kopfschmerzen	Galvanisation am Kopf und Sympathikus	20 min	Keine Angabe, insgesamt 5–13 Mal	Keine	Genesung, z. T. mit Rezidiven	[9]
Meyer	1881	1 (1 w)	Migräne	Galvanisation Schläfe-Hals	2–3 min jede Seite	2–4 Sitzungen/Woche, insgesamt 35 Mal	Keine	Geheilt, Rezidiv im Verlauf, nach 30 weiteren Sitzungen erneut geheilt	[25]
Rumpf	1881	2 (0 w)	Kopfschmerzen bei „Congestionen“	Faradisation (Faradischer Pinsel)	Keine Angabe	Alle 3 Tage, insgesamt 2 bzw. 18 Mal	Keine	Heilung	[37]
Fischer	1882	2 (2 w)	Kopfschmerzen bei „melancholischer Verstimmung“ und bei Bleichsucht	Allgemeine Faradisation	10–20 min	2–3 Sitzungen/Woche, insgesamt bis 24 Mal	Keine	Deutliche Verbesserung	[15]
Stein	1882	5 (1 w)	Neurasthenischer Kopfdruck	Franklinisation	20–30 min	Täglich, insgesamt 25–38 Mal	Keine	4 × Heilung, 1 × kein Effekt	[41]
Holst (zitiert nach Stein)	1882	8 (8 w)	4 × Neurasthenie, 4 × Migräne	Franklinisation	Keine Angabe	Keine Angabe	Keine	1 × Heilung, 6 × Teilerfolg, 1 × Verschlechterung	[41]
Tigges	1883	7 (4 w)	Kopfschmerzen im Rahmen psychischer Erkrankungen (meist Manien)	Galvanisation des Sympathikus	Keine Angabe	Keine Angabe	Keine	4 × Besserung der Kopfschmerzen, 3 × (bei „zu starkem Strom“) Zunahme von Kopfschmerzen	[47]
Stein	1886	1 (0 w)	Kopfschmerzen bei Neurasthenie	Galvanisation am Rücken und Faradisation	10 min Galvanisation und 15–17 min Faradisation	Jeweils täglich, jeweils insgesamt 14 Mal	Keine	Heilung	[42]
Eulenburg	1887	Keine Angabe	Kopfschmerz bei Neurasthenie und Zephalgien unterschiedlichster Art	Franklinisation	Keine Angabe	Keine Angabe	Keine	„sehr entschieden günstig beeinflusst“	[10]
Fischer	1887	1 (0 w)	Kopfschmerzen bei Verfolgungswahn und Halluzinationen	Galvanisation am Kopf	2 min	Täglich, insgesamt 29 Mal	Keine	Initiale Besserung, dann Wiederkehr der Kopfschmerzen	[16]
Neftel	1890	9 (6 w)	Migräne	7 × Galvanisation am Kopf, 2 × Faradisation	3 min bzw. keine Angabe	Täglich, 2 Wochen bis 3 Monate lang	Keine, aber Diskussion darüber, dass in manchen Fällen Faradisation erfolglos blieb bzw. Galvanisation symptomverstärkend wirkt	3 × Heilung, 6 × guter Teilerfolg	[28]
Sperling	1892	15 (11 w)	Kopfschmerzen verschiedener Genese (Migräne, Neuralgie, Neurasthenie)	Galvanisation am Kopf oder Hals	1–2 min	Täglich bzw. 2–3 ×/Woche	Keine, beschreibt jedoch Verschlechterung der Beschwerden nach Faradisation	8 × vollständige Genesung, 4 × Genesung mit Rezidiv im Verlauf, 3 × Teilerfolg	[40]
Morton	1899	1 (1 w)	Migräne	Elektrostatische Franklinisation (Influenzmaschine)	Keine Angabe	Keine Angabe, 6 Monate	Keine	Vollständige Genesung	[26]
Lowder	1900	„a number of patients“	Kopfschmerzen	Elektrostatische Franklinisation (Influenzmaschine)	Keine Angabe	Keine Angabe	Keine	85–90 % Heilung	[21]

**Abb. 1:**
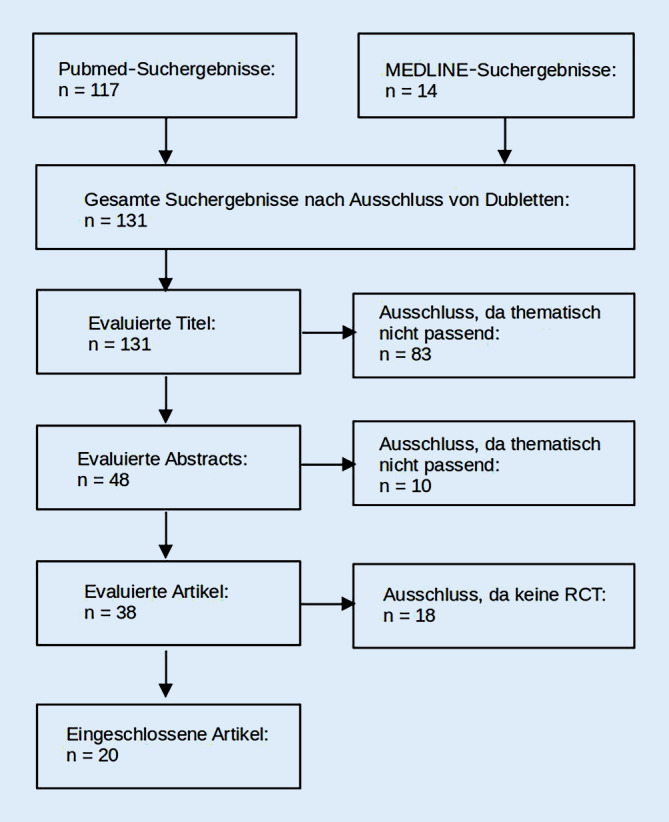
PRISMA-Flowchart bzgl. der Suchbegriffe „(tDCS AND migraine) OR (tDCS AND headache)“ in PubMed und MEDLINE am 31.10.2021

Die aktuellen Studien wurden mithilfe einer Literaturrecherche unter Verwendung der Datenbanken PubMed und MEDLINE mit dem Stichtag 31.10.2021 identifiziert. Dabei wurde die Suchbegriffkombination „(tDCS AND migraine) OR (tDCS AND headache)“ verwendet. Die so gewonnenen Suchergebnisse wurden im Titel und gegebenenfalls Abstract gescreent und alle Studien eingeschlossen, die die Anwendung von tDCS als Therapeutikum bei jedweden Kopfschmerzsyndromen untersuchten. Hierbei wurden nur klinische Studien berücksichtigt und Reviews ausgeschlossen (Abb. 1). Die Recherche und das Filtern der Ergebnisse erfolgten durch den Erstautor, bei Unklarheiten berieten sich beide Autoren bezüglich des Ein- oder Ausschlusses von Artikeln. Diese Funde wurden dann im Volltext inhaltlich erschlossen und hinsichtlich der Eckpunkte des Studiendesigns und der Ergebnisse erfasst (Tab. 2).

**Tab. 2:** Übersicht über die zeitgenössischen Publikationen zur tDCS bei Kopfschmerz

(Erst‑)Autor	Jahr	Probandenzahl	Indikation	Art der tDCS-Stimulation	Dauer der Stimulation	Frequenz/Häufigkeit der Stimulation	Kontrollgruppe/Verblindung	Erfolg	Zitat
Antal	2011	25 (23 w)	Migräne	Kathode okzipital, Anode am Scheitel	15 min	3 Tage/Woche, insgesamt 9 Mal	Kontrollgruppe mit Placebostimulation	Signifikante Abnahme der Schmerzintensität, nichtsignifikante Abnahme der Tage mit Schmerzen	[4]
Auvichayapat	2012	42	Migräne	Anode über Motorkortex	20 min	Täglich, insgesamt 20 Mal	Kontrollgruppe mit Placebostimulation	Signifikante Reduktion von Episodenfrequenz, Schmerzintensität und Analgetikaeinnahme	[5]
Dasilva	2012	13 (8 w)	Chronische Migräne	Anode über Motorkortex, Kathode supraorbital	20 min	3 ×/Woche, insgesamt 10 Mal	Kontrollgruppe mit Placebostimulation	Signifikante Reduktion der Schmerzintensität und Episodenfrequenz	[13]
Pinchuk	2013	90 (64 w)	Migräne, Spannungskopfschmerzen und posttraumatische Kopfschmerzen	Anode frontal, Kathode am Mastoid	30–45 min	1–2 ×/Woche, insgesamt 5–9 Mal	Keine	Signifikante Verbesserung bei Patienten mit Migräne und Spannungskopfschmerzen	[31]
Viganò	2013	13 (11 w)	Episodische Migräne	Anode okzipital, Kathode am Kinn	15 min	2 ×/Woche, insgesamt 16 Mal	Keine	Signifikante Reduktion der Episodenfrequenz und der Tage mit Schmerzen	[48]
Dalla Volta	2015	60	Chronische Migräne	Nicht spezifiziert	10 min	Jeden 2. Tag, insgesamt 5 Mal	Kontrollgruppe mit Placebostimulation	Nach 1 Monat bei 75 % Verbesserung, nach 2 Monaten bei 40 % anhaltende Verbesserung	[11]
Rocha	2015	19	Migräne	Kathode okzipital	Keine Angabe	Keine Angabe, insgesamt 12 Mal	Kontrollgruppe mit Placebostimulation	Signifikante Reduktion der Episodenfrequenz und -dauer	[36]
Wickmann	2015	20 (20 w)	Menstruationsassoziierte Migräne	Kathode okzipital	20 min	Täglich über 5 Tage vor Zyklusbeginn, drei Monate lang	Kontrollgruppe mit Placebostimulation	Nichtsignifikante Abnahme der Episodenfrequenz	[50]
Alhassani	2017	9 (6 w)	Chronische Migräne oder Spannungskopfschmerz	Anode über Motorkortex, Kathode supraorbital sowie transspinale DCS (Anode über HWK 10)	20 min jeweils	Täglich, insgesamt 5 Mal	Keine	Reduktion der Schmerzhäufigkeit, jedoch nicht der Schmerzintensität oder -dauer	[2]
Andrade	2017	13	Migräne	Anode über Motorkortex oder dorsolateralem präfrontalem Kortex (DLPFC)	20 min	Keine Angabe, insgesamt 12 Mal	Kontrollgruppe mit Placebostimulation und Vergleich beider Stimulationsmethoden	Signifikante Abnahme der Schmerzintensität und Zunahme der Lebensqualität, insbesondere in der DLPFC-Gruppe, zudem weniger Nebenwirkungen in DLPFC-Gruppe	[3]
Przeklasa-Muszyńska	2017	50	Migräne	Nicht spezifiziert	Keine Angabe	Keine Angabe, insgesamt 10 Mal	Kontrollgruppe mit ausschließlich pharmakotherapeutischen Interventionen	36–40 % Schmerzabnahme, deutlich mehr als in pharmakotherapeutischer Gruppe	[33]
Magis	2018	31 (9 w)	Chronische Clusterkopfschmerzen	Anode frontal (Fz), Kathode Nacken (C7)	20 min	Täglich, 4–8 Wochen	Keine	45 % signifikante Besserung, aber nach 2 Wochen Rezidive	[22]
Ahdab	2019	43 (35 w)	Episodische Migräne	Kathode okzipital, Anode supraorbital	20 min	Täglich, insgesamt 3 Mal	Kontrollgruppe mit Placebostimulation	Signifikante Reduktion der Tage mit Schmerzen, Analgetikaeinnahme und Schmerzintensität	[1]
Park	2019	30 (21 w)	Zervikogener Kopfschmerz	Anode und Kathode über Motorkortex, kombiniert mit Bewegungsübungen	Keine Angabe	Keine Angabe; 4 Wochen	Kontrollgruppe, die nur mit Bewegungsübungen behandelt wurde	Signifikant stärkere Reduktion der Schmerzen im Vergleich zur Kontrollgruppe	[29]
Dalla Volta	2020	45 (30 w)	Chronische Migräne	Kathode über thermografisch identifizierter kältester Stelle am Kopf	15 min	Täglich für 5 Tage, anschließend einmalig nach 1 Monat	Verblindete Kontrollgruppe mit Placebostimulation	Signifikante Verbesserung sämtlicher Parameter (Anfallsfrequenz, Tage mit Schmerzen, Schmerzmitteleinnahme) auch nach 120 Tagen	[12]
Grazzi	2020	135	Migräne und medikamentös induzierter Kopfschmerz	Entweder Kathode oder Anode über Motorkortex	20 min	Täglich, insgesamt 5 Mal	Kontrollgruppe mit Placebostimulation und Vergleich beider Stimulationsmethoden	Keine signifikante Veränderung im Vergleich zur Placebogruppe	[17]
Mansour	2020	18 (17 w)	Medikamentös induzierter Kopfschmerz	Anode auf präfrontalem Kortex oder Kathode okzipital	20 min	Täglich, insgesamt 3 Mal	Kontrollgruppe mit Placebostimulation und Vergleich beider Stimulationsmethoden	Signifikante Reduktion der Tage mit Schmerzen, jedoch nur bei okzipitaler Stimulation Anhalten des Effekts über 14 Tage und Reduktion der Analgetikaeinnahme	[23]
Pohl	2020	23 (12 w)	Episodische Migräne	Anode okzipital, Kathode am Scheitel	20 min	Täglich, insgesamt 28 Mal	Kontrollgruppe mit Placebostimulation	Signifikante Reduktion der Tage mit Schmerzen	[32]
Rahimi	2020	45 (40 w)	Migräne, episodisch und chronisch	Kathode am Kopf (C4), Anode auf dem Arm	20 min	3 ×/Woche, dann seltener, insgesamt 22 Mal	Kontrollgruppe mit Placebostimulation	Signifikante Verbesserung sämtlicher Parameter (Anfallsfrequenz, Schmerzintensität, Schmerzdauer)	[34]
De Icco	2021	20 (16 w)	Migräne und medikamentös induzierter Kopfschmerz	Anode über Motorkortex (C3 oder C4), Kathode supraorbital	20 min	Täglich, insgesamt 5 Mal	Kontrollgruppe mit Placebostimulation	Signifikante Reduktion der Tage mit Schmerzen	[14]

## Ergebnisse

Insgesamt wurden infolge unserer Literaturrecherche 15 historische Veröffentlichungen identifiziert, die anhand von Kasuistiken oder Fallserien von der Elektrotherapie bei Kopfschmerzen berichteten. Die umfangreichste Stichprobe beinhalteten Arthur Sperlings „Elektrotherapeutische Studien“ [40] mit 15 Patienten, jedoch behandelten vier der eingeschlossenen Publikationen dagegen nur eine einzelne Patientenkasuistik, drei Werke gaben wiederum keine genaue Angabe der Patientenzahl. Setzt man voraus, dass die Studien ohne Angabe einer Patientenzahl mindestens die Behandlung eines Kranken umfassen müssen, ergibt sich eine Gesamtzahl von 59 Patienten. Am häufigsten (siebenmal) wurde die Galvanisation angewendet, aber auch die anderen beiden beliebten Stimulationsmethoden – die Franklinisation und die Faradisation – sind mit fünf bzw. vier Studien präsent. Dabei beschreibt der russisch-amerikanische, seit 1868 in New York praktizierende Nervenarzt William Basil Neftel sowohl die Anwendung der Faradisation als auch der Galvanisation [28]. Fünf Studien behandelten Patienten mit Migräne, der „neurasthenische Kopfschmerz“ ist ebenfalls in fünf Studien Indikation für die Therapie. In drei Arbeiten waren die Kopfschmerzen Begleitsymptome anderweitiger psychiatrischer Erkrankungen, zwei widmen sich dem „congestiven Kopfschmerz“. Dabei ist zu erwähnen, dass oft Patienten mit unterschiedlicher Genese des Kopfschmerzes in einem Werk behandelt werden oder teilweise nicht auf die Genese oder Einteilung des Kopfschmerzes eingegangen wurde. Die eingesetzte Dauer der Stimulation variiert von einer Minute bis 30 min, wobei circa zwei Minuten und circa 20 min am häufigsten repräsentiert sind. Die jeweiligen Anzahlen der insgesamt durchgeführten Behandlungen lagen zwischen zwei bis über 40, wobei – sofern eine Gesamtanzahl der Behandlungen genannt wird – diese sich meist im Bereich von 15 bis 30 Anwendungen bewegt. Hinsichtlich der Behandlungsresultate der Elektrotherapie dominieren Berichte von Genesung oder zumindest sehr guten Teilerfolgen. Es werden insgesamt lediglich vier Patientenfälle aufgeführt, bei denen die Behandlung auf die Kopfschmerzen keinen Effekt hatte oder gar zu einer Verschlechterung der Symptomatik geführt hatte. Vier Veröffentlichungen beschreiben allerdings eine Reihe von Fällen, bei denen es im Verlauf zu Rezidiven kam, die dann meist jedoch gut auf eine Wiederaufnahme der Behandlung ansprachen. Keine der historischen Publikationen operierte mit einer Kontrollgruppe, es wurden höchstens Vergleiche von verschiedenen Methoden der elektrotherapeutischen Anwendungen durchgeführt.

Bei der Suche nach kontemporären Studien wurden 20 Publikationen identifiziert. Diese umfassten von neun bis 135 Probanden, wobei die meisten Stichproben sich in der Größenordnung von 13 bis 50 Probanden bewegten. In Summe hatten die eingeschlossenen Studien 744 Probanden. Obwohl im Rahmen der Literaturrecherche gezielt nach allen Kopfschmerzformen gesucht wurde, behandelte ein erheblicher Großteil der Veröffentlichungen (17 Studien) die Kopfschmerzform der Migräne – fünf davon explizit die chronische Form, fünf explizit die episodische Form, zwei in der Kombination mit medikamentös induziertem Kopfschmerz, alle restlichen Studien machten keine Angabe über die Migräneform. Nur in zwei Studien wurden Spannungskopfschmerzen untersucht, jeweils eine Publikation widmete sich Clusterkopfschmerzen, zervikogenen Kopfschmerzen und dem ausschließlichen medikamentös induzierten Kopfschmerz. Da als Stimulationsart die tDCS unser Eingangssuchkriterium war, ist sie bei allen 20 Studien die eingesetzte Therapiemethode. Allerdings ist eine große Varianz an Stimulationsformen hinsichtlich der Polarität der Stimulation (kathodal oder anodal) und der Positionierung der Elektroden repräsentiert. Die häufigsten Stimulationselektrodenpositionen waren über dem Motorkortex (zehn Studien, sieben davon anodale Stimulation und drei kathodal) und okzipital (sieben Studiendesigns, fünf davon kathodal und zwei anodal). Darüber hinaus waren aber auch die (prä-)frontale Stimulation und die Positionierung über dem dorsolateralen präfrontalen Kortex sowie die spinale Positionierung und diejenige über der thermografisch identifizierten kältesten Stelle am Kopf vertreten. Zwei Studien machten keine Angabe bezüglich der Stimulationsorte. Insgesamt wurde 14 Mal anodal und neun Mal kathodal stimuliert. Hinsichtlich der Anwendungsdauer zeichnet sich eine größere Vereinheitlichung ab, mit zwölf Studien, die 20 min Stimulationsdauer einsetzten, die restlichen verteilten sich auf Zeiträume zwischen zehn und 45 min. Bezüglich der Gesamtanzahl der Behandlungen gab es jedoch eine erhebliche Streuungsbreite von drei Mal bis über 50 Mal (dabei waren zehn bis 20 Anwendungen am häufigsten vertreten). Vier Studien arbeiteten ohne Kontrollgruppe, 14 nutzten (meist sogar doppelt verblindet) die Placebostimulation als Kontrollgruppe, zwei Veröffentlichungen hatten Kontrollgruppen mit ausschließlich physiotherapeutischer bzw. pharmakologischer Behandlung zum Vergleich. 13 Publikationen berichten von einer erheblichen Symptomverbesserung, sechs zumindest von einer Besserung einzelner Parameter oder nur bei einem Teil der Probanden. Eine Studie fand keine Verbesserung der Kopfschmerzen durch tDCS im Vergleich zur Kontrollgruppe, dabei erscheint es interessant zu vermerken, dass dies die Studie mit den meisten Probanden war [17]. In drei Aufsätzen wurden explizit das Nachlassen der Wirkung über die Zeit und Rezidive erwähnt.

## Diskussion

Die Ergebnisse sowohl der historischen elektrotherapeutischen Arbeiten als auch der Publikationen zur modernen tDCS als ihrem kontemporären Erbe legen nahe, dass mit der medizinischen Applikation von Strom vielversprechende, wirksame Ansätze zur Behandlung von diversen Kopfschmerzsyndromen verbunden sein könnten. Allerdings wird die Aussagekraft der Studien durch ihre bisher vorliegenden eher kleinen Stichproben geschmälert. Insbesondere die historischen Quellen basierten oft auf der Behandlung einzelner oder weniger Patienten, da es der damaligen, eben nicht umfangreiche empirische und evaluierbare Daten sammelnden Forschungsmethodik entsprach, gerade bei neuartigen Therapiemethoden detailliert einzelne Behandlungsgeschichten und -erfolge zu berichten. Darauf, dass hierbei vielfach ausschließlich die Erfolge und nicht die Misserfolge dokumentiert und veröffentlicht wurden, weist Neftel 1890 sogar selbst direkt hin: „Da ein einziger positiver Fall mehr Interesse darbietet, als zahlreiche negative, so führe ich beispielsweise den folgenden an“ [28, S. 133]. Doch auch die kontemporären Studien wurden häufig mit wenig Patienten durchgeführt. Alhassani et al. präsentieren die Daten von nur neun [2], andere Autoren bringen Daten von nur 20 bis 30 Probanden bei. Was an dieser Stelle auch nicht unerwähnt bleiben darf, ist die Tatsache, dass die Publikation mit der deutlich größten Probandenanzahl auch diejenige ist, die als einzige keinerlei Wirksamkeit der tDCS auf Kopfschmerzen nachweisen konnte [17]. Während die Ärzte des 19. Jahrhunderts durchgehend ohne Kontrollgruppen arbeiteten, war die Frage von Placebowirkung und Heilung durch „Suggestion“ im damaligen wissenschaftlichen Diskurs doch gerade erst neu aufgeworfen worden und heiß umstritten [43], so nutzt nun eine überwiegende Mehrheit der modernen Studien verblindete Kontrollgruppen mit Placebostimulationen, um gerade den Effekt einer Symptomlinderung durch den Glauben an die Therapie oder andere einflussnehmende Variablen herausrechnen zu können.

Beim Vergleich aller analysierten Arbeiten fällt sofort auf, dass es sowohl im 19. Jahrhundert als auch jetzt wieder eine erstaunlich große Heterogenität in der technischen und klinischen Durchführung der Stimulationsbehandlung gibt. Wurden in den historischen Publikationen noch verschiedene Arten der elektrischen Stimulation (Galvanisation, Franklinisation, Faradisation) angewandt, hat sich in der kontemporären Medizin zwar die tDCS als maßgeblich verbreitetste Form der Stromapplikation herausgebildet, aber die Polarität der Stimulation (anodal oder kathodal) und die Positionierung der Elektroden (frontal, Motorkortex, okzipital) variieren von Studie zu Studie erheblich. Trotz dieser vielfältigen Stimulationstechniken und der ihnen zugrunde liegenden Ansätze und Konzepte zur Möglichkeit der Schmerzmodulation werden dabei aber nahezu durchgehend Erfolge berichtet.

Bei dem Versuch, die historischen und modernen Arbeiten systematisch miteinander zu vergleichen, offenbarte sich uns das Problem des Wandels der verschiedenen Theorien zur Ätiologie des Kopfschmerzes und die sich daraus ergebende Klassifikation von Kopfschmerzsyndromen. Unterscheidet man heute zwischen Migräne, Spannungskopfschmerzen, Clusterkopfschmerzen, zervikogenen Kopfschmerzen und dem Sonderfall des medikamentös induzierten Kopfschmerzes, waren die Kategorien im 19. Jahrhundert durchaus anders. Die Migräne war als diagnostische Entität bereits damals etabliert [38], jedoch waren „neurasthenischer Kopfschmerz“ und „congestiver Kopfschmerz“, dem die Genese eines Blutstaus im Kopf zugeschrieben wurde, in der medizinischen Literatur der Epoche ebenfalls verbreitete Diagnosen, denen man heute schwer ein entsprechendes Pendant zuweisen kann. Auffällig ist des Weiteren bei den kontemporären Studien, dass sich ein Großteil ausschließlich mit der Migräne als Kopfschmerzsyndrom befasst, obgleich Kopfschmerzen, die gemäß der heutigen Klassifikation dem Spannungskopfschmerztyp zugeschrieben werden, verbreiteter sind und deshalb in Summe ein größeres Ausmaß an Behinderung verursachen [46]. Was der Hintergrund dieses Fokus der Forschung auf die Migräne ist, bleibt unklar.

Eine Fragestellung, auf die sowohl die historischen als auch die zeitgenössischen Quellen nur wenig eingehen, ist die nach der langfristigen Wirksamkeit elektrotherapeutischer Methoden. Der Beobachtungszeitraum betrug im 19. Jahrhundert oft nur die Dauer der Behandlung und auch in den modernen Studien wurden die Symptome oft nur wenige Wochen bis Monate nach Beendigung des Behandlungszyklus erhoben. Einige Arbeiten erwähnten zwar Rezidive oder ein Nachlassen der schmerzstillenden Wirkung im Verlauf der Zeit, eine systematische Betrachtung dieser Frage fehlte und fehlt jedoch damals wie heute.

An dieser Stelle sollen auch die Limitationen unserer Arbeit erwähnt werden. Sowohl für die Identifikation der historischen als auch für die Identifikation der kontemporären Quellen wurde jeweils nur ein Ausgangspunkt für die Recherche genutzt – „Schmidts Jahrbücher der in- und ausländischen gesammten Medicin“ respektive PubMed und MEDLINE. Zudem war die Publikationsauswahl (mit der Ausnahme von Calonius’ Studie [9], für die aus dem Finnischen eine maschinelle Übersetzung angefertigt wurde) auf deutsch- und englischsprachige Werke beschränkt. Des Weiteren erfolgte bei den kontemporären Studien eine Beschränkung auf tDCS als untersuchte Stimulationsmethode, welche zwar die verbreitetste, aber mitnichten die einzige Form der modernen Elektrotherapie ist [30]. Unser Motiv liegt darin begründet, dass die tDCS den historischen Techniken der Stromverabreichung am nächsten kommt und wir eine Kontinuität dieser Therapiemethode und deren Problematiken in der Schmerzmedizin sehen und vermitteln wollten. Somit erhebt diese Arbeit aber keineswegs den Anspruch auf eine umfassende Wiedergabe sämtlicher Literatur auf dem Feld der Elektrotherapie der Kopfschmerzen. Viel eher war es unsere Absicht, einen Einblick in die Erforschung dieses Themas in der ersten Blütezeit der Elektrotherapie im 19. Jahrhundert sowie heute zu geben und diese oft voneinander getrennt gedachten und betrachteten Felder zusammenzuführen und zu vergleichen.

## Fazit für die Praxis

Elektrotherapeutische Ansätze zur Behandlung von Kopfschmerzen blicken auf eine über 140-jährige Geschichte zurück und werden aktuell wieder vielfach erforscht. Die Ergebnisse ihrer Anwendung präsentieren sich in den Artikeln damals wie heute als vielversprechend, allerdings arbeiten Studien, die sich dieser Therapieform widmen, oft mit einer geringen Fallzahl. Zudem fällt eine große Heterogenität der Stimulationsmethodiken auf. Dazu kommt, dass andere Kopfschmerzsyndrome als Migräne insbesondere in der kontemporären Forschung wenig Beachtung finden. Um mit größerer Gewissheit über den Nutzen dieses Behandlungsansatzes sprechen zu können, aber auch um die bestmöglich geeignete Stimulationsform zu identifizieren, sind weitere, umfangreichere Studien vonnöten. Die Beschäftigung mit der Elektrotherapie des 19. Jahrhunderts würdigt dabei nicht nur den Ausgangspunkt dieses medizinischen Felds, sondern kann auch hilfreiche Impulse für die aktuelle Forschung geben.
